# Hedging for the Regime-Switching Price Model Based on Non-Extensive Statistical Mechanics

**DOI:** 10.3390/e20040248

**Published:** 2018-04-03

**Authors:** Pan Zhao, Jian Pan, Benda Zhou, Jixia Wang, Yu Song

**Affiliations:** 1 College of Finance and Mathematics, West Anhui University, Lu’an 237012, China; 2 Financial Risk Intelligent Control and Prevention Institute of West Anhui University, Lu’an 237012, China; 3 College of Mathematics and Computer Science, Gannan Normal University, Ganzhou 341000, China; 4 School of Mathematics and Information Sciences, Henan Normal University, Xinxiang 453002, China; 5 School of Economics and Management, Nanjing University of Science and Technology, Nanjing 210094, China

**Keywords:** non-extensive statistics, hedging, risk-minimizing approach, Föllmer–Schweizer decomposition

## Abstract

To describe the movement of asset prices accurately, we employ the non-extensive statistical mechanics and the semi-Markov process to establish an asset price model. The model can depict the peak and fat tail characteristics of returns and the regime-switching phenomenon of macroeconomic system. Moreover, we use the risk-minimizing method to study the hedging problem of contingent claims and obtain the explicit solutions of the optimal hedging strategies.

## 1. Introduction

Describing asset price changes accurately is a basis for pricing and risk management of financial derivatives. Usually, a geometric Brownian motion was employed to portray the changes of asset prices [1,2,3]. The assumption that the asset price obeys the geometric Brownian motion means that the distribution of the asset returns is normal. However, many empirical results have shown that the distribution of the yield rate has the characteristics of high peak and fat tail, it is not a normal distribution [4,5,6].

Fortunately, in the field of non-extensive statistics, several scholars have found that the Tsallis distribution derived from non-extensive Tsallis entropy can depict the characteristics of high peak and fat tail of returns. For example, Kozuki found that the Tsallis distribution qualitatively agrees with the fat-tailed data of foreign exchange market [7]. Tsallis et al. found that the distribution of stock yield obeys a Tsallis distribution [8]. Moreover, Borland proposed an option pricing model, in which the underlying stock price was driven by a stochastic process constructed by the maximizing non-extensive Tsallis entropy. Furthermore, he obtained the formula for pricing European options [9]. Those studies showed that the Tsallis non-extensive statistical theory is better than the classical extensive statistical method in the financial field.

In addition, the above asset price model is a short-term microcosmic model and ignores the impact of long-term macro-economy, such as the adjustment of the economic structure, the change of the market system and the cycle of the business cycle. However, several empirical studies have shown that there is a phenomenon of regime switching in the long-term financial market. For example, Mary proposed a regime-switching lognormal model and found that the fitting of stock prices is more accurate using the regime-switching model than other common econometric models [10]. In recent years, the pricing and the optimal investment of financial derivatives based on the regime-switching model have been paid more and more attention by financial scholars. Elliott considered the pricing problem of European options when the risky asset was driven by the regime-switching geometric Brownian motion [11]. Chi studied the pricing problem of barrier and lookback options when the underlying assets were driven by the regime-switching jump-diffusion process [12]. Yiu and zhu proposed an optimal portfolio selection model with a value-at-Risk constraint. In the model the risky assets were driven by the regime-switching geometric Brownian motion [13,14]. Zhang considered the mean-variance portfolio selection problem when the risky assets were driven by the regime-switching geometric Brownian motion [15].

In this study, to describe the movement of asset prices accurately, we employ the Tsallis entropy distribution and the semi-Markov process to establish an asset price model. The model can depict the characteristics of high peak and fat tail of asset returns and the regime-switching phenomenon of macroeconomic system. Moreover, we use the risk-minimizing method to study the hedging problem of contingent claims and obtain the explicit solutions of the optimal hedging strategies.

The paper is organized as follows. In Section 2, we use the non-extensive statistical theory to establish the asset price model that can depict the phenomenon of high peak and fat tail of asset returns. In Section 3, we embed the semi-Markov process into the above model so that it can be developed to portray the macroeconomic impact. In Section 4, under the framework of minimizing risk, the hedging problem of financial derivatives is studied. Furthermore, the explicit solutions to the optimal hedging problem are obtained by the use of the minimal martingale measure method and the Föllmer–Schweizer decomposition technique. In the last Section 5, the summary of the paper is given.

## 2. Asset Price Model

It is well known that the price movement of risky assets is affected by many interrelated factors, which brings about the fat-tail characteristics of return distribution. However, the asset price driven by the classical geometric Brownian motion is normal, which cannot describe the fat-tail characteristics of returns. Thus, to accurately fit the price changes of risky assets, we employ a stochastic process derived from the non-extensive statistical theory to replace the classical geometric Brownian motion (see [9]). Furthermore, the price process of risky asset can be written as

(1) dS(t)=μS(t)dt+σS(t)dΩ(t)

where

(2) $$d\Omega \left(t\right)=P{\left(\Omega ,t\right)}^{\frac{1-q}{2}}dW\left(t\right)$$

W(t) is a Wiener process. P(Ω,t) is a probability density function satisfying the maximum Tsallis entropy. It is given by:

(3) $$P\left(\Omega ,t\right)=\frac{1}{z(t)}{\left(1-\beta \left(t\right)\left(1-q\right)\Omega^{2}\right)}^{\frac{1}{1-q}}$$

with

(4) $$z\left(t\right)={\left(\left(2-q\right)\left(3-q\right)ct\right)}^{\frac{1}{3-q}}$$

(5) $$\beta \left(t\right)=c^{\frac{1-q}{3-q}}{\left(\left(2-q\right)\left(3-q\right)t\right)}^{-\frac{2}{3-q}}$$

and

(6) $$c=\frac{\pi }{q-1}\frac{\Gamma^{2}\left(\frac{3-q}{2(q-1)}\right)}{\Gamma^{2}\left(\frac{1}{q-1}\right)}$$

In the limit q→1, the above probability density function degenerates into a normal distribution, which is the case of the classical geometric Brownian motion. However, when $1<q<\frac{5}{3}$, it has a sharper peak and thicker tails than a normal distribution. Hence, the asset price model can depict the characteristics of high peak and fat tail of returns and accurately fit the changes of asset prices.

## 3. Regime-Switching Asset Price Model

Let T be a positive number, denoted a finite time horizon. Let $\left(\Omega ,F,{\left(F_{t}\right)}_{0\leq t\leq T},P\right)$ be a probability space. The above probability space satisfies the usual conditions of right-continuity and completeness.

In the market, we suppose that there are two underlying assets: a risk-free bond and a risky stock. The price process of the risk-free bond is written as follows

(7) $$\left\{\begin{array}{c} dB(t)=rB(t)dt,t\in [0,T]\\ B\left(0\right)=B_{0} \end{array}\right.$$

where *r* is a positive constant called the risk-free interest rate. The price process of the risky stock is given by

(8) $$dS\left(t\right)=\mu \left(Y_{t}\right)S\left(t\right)dt+\sigma \left(Y_{t}\right)S\left(t\right)d\Omega \left(t\right)$$

where

(9) $$d\Omega \left(t\right)=P{\left(\Omega ,t\right)}^{\frac{1-q}{2}}dW\left(t\right)$$

W(t)(0≤t) is a Wiener process. P(Ω,t) is the Tsallis distribution of index *q*, which can depict the characteristics of high peak and fat tail of the stock returns. $Y_{t}$ is a semi-Markov process at the phase space $\left(Y,\Upsilon \right),Y_{t}=Y_{\kappa (t)},\kappa \left(t\right)=\max\left\{n:\tau_{n}\leq t\right\},\tau_{n}=\sum_{k=1}^{n}\theta_{k}$. Suppose that $P\left\{\omega :Y_{n+1}\in \;B,\theta_{n+1}\leq t|Y_{n}=y\right\}=P\left(y,B\right)\times V_{y}\left(t\right),B\in \Upsilon ,\;y\in Y.\;V_{y}\left(t\right)$ is a differentiable function of *t* and $v_{y}\left(t\right)=\frac{dV_{y}\left(t\right)}{dt}$. This semi-Markov process can describe regime switching of the macro economy. In addition, we suppose that the Wiener process $W_{t}$ and the semi-Markov process $Y_{t}$ are independent, a semi-Markov process. The filtration $F_{t}$ is generated by the random processes $W_{t}$ and $Y_{t}$.

## 4. Risk-Minimizing Hedging

Now, we consider the hedging problem of the European call option. That is, we try to hedge against the contingent claim by means of portfolio strategies. Let *K* be a strike price and *T* be a maturity date. Then, at the maturing time *T*, the payment of the European call option is $H={\left(S\left(T\right)-K\right)}^{+}$. The hedging portfolio strategy defines a portfolio with the number of units of the stock $\alpha_{t}$ and the number of units of the bond $\beta_{t}$. Let $\varphi =(\alpha_{t},\beta_{t})$ represent a portfolio strategy and satisfy

(10) $$E\int_{0}^{T}\alpha_{t}^{2}\sigma^{2}\left(Y_{t}\right)P^{1-q}S_{t}^{2}dt+E[\int_{0}^{T}|\alpha_{t}||\mu \left(Y_{t}\right){\left|S_{t}dt\right]}^{2}<+\infty$$

Then, the discounted value process $V_{t}\left(\varphi \right)$ of the portfolio can be written as follows

(11) $$V_{t}\left(\varphi \right)=\alpha_{t}S_{t}^{*}+\beta_{t}$$

where the discounted process $S_{t}^{*}=\frac{S_{t}}{B_{t}}$.

Thus, the cumulative cost process $C_{t}\left(\varphi \right)$ can be given as follows

(12) $$C_{t}\left(\varphi \right)=V_{t}\left(\varphi \right)-\int_{0}^{t}\alpha_{v}dS_{v}^{*},\;0\leq t\leq T.$$

The $C_{t}\left(\varphi \right)$ is the total cost generated by the portfolio strategy φ over the interval [0,t], which is from trading because of the fluctuations of the asset price process $S_{t}$ and not due to the transaction cost (see [16]). Since the payment of the European call option $H={\left(S\left(T\right)-K\right)}^{+}$ is $F_{T}$-measurable and the strategy $\beta_{t}$ is adapted, the hedging portfolio policy $\varphi =(\alpha_{T},\beta_{T})$ with $V_{T}\left(\varphi \right)=H={\left(S\left(T\right)-K\right)}^{*}$P−a.s. exists. Thus, when the cost process $C_{t}\left(\varphi \right)$ is square-integrable, the risk of the hedging portfolio strategy φ can be defined as follows

(13) $$R_{t}\left(\varphi \right)=E\left[{\left(C_{T}\left(\varphi \right)-C_{t}\left(\varphi \right)\right)}^{2}|F_{t}\right],\;0\leq t\leq T.$$

Then, the problem of the optimal hedging portfolio strategy becomes an optimization problem

(14) $$\min_{\varphi }R_{t}\left(\varphi \right)=\min_{\varphi }E\left[{\left(C_{T}\left(\varphi \right)-C_{t}\left(\varphi \right)\right)}^{2}|F_{t}\right],\;0\leq t\leq T.$$

From the literature [17], we know that, in a complete market, we need to construct a new probability measure $P^{*}$ and the new probability measure is equivalent to the original probability measure *P*. Under the new probability measure $P^{*}$, we can employ a martingale to represent the original asset price process. Then, we can replicate the contingent claim by a self-financing policy. That is, the risk $R_{t}\left(\varphi \right)$ of the contingent claim *H* can be reduced to zero by a suitable self-financing portfolio policy φ. However, in an incomplete market, this is no longer possible. In this paper, the market is incomplete owing to the embedded semi-Markov process $Y_{t}$. Thus, we cannot find a self-financing portfolio strategy φ to reduce the risk $R_{t}\left(\varphi \right)$ to zero. However, we know that the existence of the optimal risk-minimizing hedging portfolio policy is equivalent to the existence of the Föllmer–Schweizer decomposition of the contingent claim $H={\left(S\left(T\right)-K\right)}^{+}$ (see [18]). Furthermore, the Föllmer–Schweizer decomposition of the contingent claim $H={\left(S\left(T\right)-K\right)}^{+}$ can be written as follows

(15) $$H=H_{0}+\int_{0}^{T}\alpha_{v}^{H}dS_{v}+L_{T}^{H},\;\left(P-a.s.\right)$$

where $H_{0}\in L^{2}\left(F_{0},P\right)$, $\alpha^{H}\in L^{2}\left(S\right)$, and $L_{T}^{H}\in M_{0}^{2}\left(P\right)$ is a square-integrable martingale orthogonal to *S*. Then, the optimal risk-minimizing hedging portfolio policy $\varphi^{*}=\left(\alpha^{H},H_{0}+L^{H}\right)$. Thus, we can derive the optimal risk-minimizing hedging portfolio policy by means of the Föllmer–Schweizer decomposition approach. To obtain the Föllmer–Schweizer decomposition, we will employ the minimal martingale measure method introduced in the literature [17]. Let

(16) $$p^{T}=\exp\left(-\int_{0}^{T}\frac{\mu (Y(v))}{\sigma \left(Y\left(v\right)\right)P\frac{1-q}{2}\left(v\right)}dW\left(t\right)-\frac{1}{2}\int_{0}^{T}\frac{\mu^{2}\left(Y\left(v\right)\right)}{\sigma^{2}\left(Y\left(v\right)\right)P\frac{1-q}{2}\left(v\right)}dv\right)$$

Then, we can define the minimal martingale probability measure $P^{*}$ equivalent to the original probability measure *P* as follows

(17) $$\frac{dP^{*}}{dP}=p^{T}$$

Below, it is our work to find the Föllmer–Schweizer decomposition under the equivalent minimal martingale probability measure $P^{*}$ as follows

(18) $$E^{*}\left(H|F_{t}\right)=E^{*}H+\int_{0}^{t}\alpha_{v}^{*H}dS_{v}+L_{t}^{*H}$$

Let the jump probability measure of the semi-Markov process $Y_{t}$ notate as follows

(19) $$\lambda \left(\left[0,t\right]\times A\right)=\sum_{k=0}^{+\infty }I\left(Y_{n}\in A,\tau_{n}\leq t\right)$$

Then, we can obtain the dual predictable projection measure (see [19]) for the probability measure λ as follows

(20) $$\tilde{\lambda }\left(dy,dt\right)=\sum_{k=0}^{+\infty }I\left(\tau_{n}<t\leq \tau_{n+1}\right)\frac{P\left(Y_{n},dy\right)v_{Y_{n}}\left(t\right)}{1-V_{Y_{n}}\left(t\right)}dt$$

and the Föllmer–Schweizer decomposition of the contingent claim $H={\left(S\left(T\right)-K\right)}^{+}$ as follows

(21) $$E^{*}\left(H|F_{t}\right)=E^{*}H+\int_{0}^{t}\alpha_{v}^{*H}dS_{v}+\int_{0}^{t}\int_{\Upsilon }M\left(v,y\right)\left(\lambda -\tilde{\lambda }\right)dvdy$$

Hence, the optimal risk-minimizing hedging portfolio strategy is

(22) $$\varphi^{*}\left(t\right)=\left(E_{t}^{*H},E^{*}\left(H|F_{t}\right)-\alpha_{t}^{*H}S_{t}\right)$$

To obtain the exact representation of the optimal risk-minimizing hedging portfolio strategy $\varphi^{*}\left(t\right)$, we need consider that the following differential equation exists a solution.

**Lemma** **1.** *Let the function g(x) satisfy $|g\left(x\right)|\;\leq \;c\times {\left(1+|x|\right)}^{n},\;0\leq n$. Then, the following differential equation*

(23) $$\left\{\begin{array}{c} \frac{\partial h(t,x,y)}{\partial t}+\frac{1}{2}\sigma^{2}\left(x\right)\times x^{2}P^{1-q}\frac{\partial h(t,x,y)}{\partial x^{2}}+\frac{v_{y}\left(t\right)}{1-V_{y}\left(t\right)}\int_{\Upsilon }P\left(y,dz\right)\left[h\left(t,x,z\right)-h\left(t,x,y\right)\right]=0\\ h(T,x,y)=g(x) \end{array}\right.$$

*has a solution as follows*

(24) $$h\left(t,x,y\right)=Eg\left({\hat{S}}_{T-t}^{x,y}\right)$$

*where*

(25) $$\hat{S}\left(t\right)=\hat{S}\left(0\right)\exp\left\{-\frac{1}{2}\int_{0}^{t}\sigma^{2}\left(Y_{S}\right)P^{1-q}ds+\int_{0}^{t}\sigma \left(Y_{S}\right)P^{\frac{1-q}{2}}dW\left(s\right)\right\}$$

**Proof** **of Lemma 1.** Firstly, we can easily verify that the solution of the following Equation (26)

(26) $$d\hat{S}\left(t\right)=\sigma \left(Y_{t}\right)P^{\frac{1-q}{2}}\hat{S}\left(t\right)dW\left(t\right)$$

is

(27) $$\hat{S}\left(t\right)=\hat{S}\left(0\right)\exp\left\{-\frac{1}{2}\int_{0}^{t}\sigma^{2}\left(Y_{S}\right)P^{1-q}ds+\int_{0}^{t}\sigma \left(Y_{S}\right)P^{\frac{1-q}{2}}dW\left(s\right)\right\}$$

This is because, using the It$\bar{o}$ formula, we can get

(28) $$d\ln\hat{S}\left(t\right)=-\frac{1}{2}\sigma^{2}\left(Y_{t}\right)P^{1-q}dt+\sigma \left(Y_{t}\right)P^{\frac{1-q}{2}}dW\left(t\right)$$

Integrating both sides of Equation (28), we can obtain

(29) $$\ln\hat{S}\left(t\right)-\ln\hat{S}\left(0\right)=-\frac{1}{2}\int_{0}^{t}\sigma^{2}\left(Y_{S}\right)P^{1-q}ds+\int_{0}^{t}\sigma \left(Y_{S}\right)P^{\frac{1-q}{2}}dW\left(s\right)$$

Calculating exponential function both sides of Equation (29), we obtain Equation (27). Substituting Equation (27) into Equation (24), we obtain

(30) $$\begin{array}{ccc} h(T-t,x,y) & = & Eg({\hat{S}}_{t}^{x,y}) \end{array}$$

(31) $$\begin{array}{cc} = & \int g\left(z\right)z^{-1}u\left(z;t,x,y\right)dz \end{array}$$

where

(32) $$\begin{array}{ccc} u(z;t,x,y) & = & \int \varphi \left(\eta ,\ln\frac{z}{x}+\frac{1}{2}\eta \right)F_{d}^{y}\left(d\eta \right) \end{array}$$

(33) $$\begin{array}{cc} = & E\varphi (x_{t}^{y},\ln\frac{z}{x}+\frac{1}{2}x_{t}^{y}) \end{array}$$

and $\varphi \left(t,x\right)=\frac{1}{\sqrt{2\pi t}}e^{-\frac{x^{2}}{2t}}$, $F_{t}^{y}$ is the distribution function of $Z_{t}^{y}=\int_{0}^{t}\sigma^{2}\left(Y_{S}^{y}\right)P^{1-q}ds$. Considering the following equation

(34) $$\left\{\begin{array}{c} \phi_{z}\left(t,\xi ,y\right)=\sigma^{2}\left(y\right)P^{1-q}\phi_{z}\left(t,\xi ,y\right)+\frac{v_{y}\left(t\right)}{1-V_{y}\left(t\right)}\int_{\Upsilon }P\left(y,dz\right)\left[\phi \left(t,\xi ,z\right)-\phi \left(t,\xi ,y\right)\right]+\phi_{t}\left(t,\xi ,y\right)\\ \phi \left(0,\xi ,y\right)=\varphi (\xi -\ln\left(\frac{x}{y}\right),\frac{\ln(\frac{x}{y})+\xi }{2}) \end{array}\right.$$

and using the It$\bar{o}$ formula, we can obtain

(35) $$\phi \left(t,\xi ,y\right)=E[\varphi \left(\xi +Z_{t}^{Y}-\ln\left(\frac{x}{y}\right),\frac{\ln\left(\frac{x}{y}\right)+\xi +Z_{t}^{Y}}{2}\right)]$$

Thus, we have

(36) $$u\left(z;t,x,y\right)=\phi \left(\ln\frac{z}{x},t,y\right)$$

Substituting Equations (35) and (36) into Equation (34), we obtain

(37) $$\left\{\begin{array}{c} u_{t}\left(z;t,x,y\right)=\sigma^{2}\left(y\right)P^{1-q}E\left[\varphi_{t}\left(Z_{t}^{y},\ln\left(\frac{z}{x}\right)+\frac{Z_{x}^{y}}{2}\right)+\frac{1}{2}\varphi_{x}\left(Z_{t}^{y},\ln\left(\frac{z}{x}\right)+\frac{Z_{x}^{y}}{2}\right)\right]\\ +\frac{v_{y}\left(t\right)}{1-V_{y}\left(t\right)}\int_{\Upsilon }P\left(y,dz\right)\left[u\left(t,x,z\right)-u\left(t,x,y\right)\right]+2u_{t}\left(z;t,x,y\right)\\ \phi \left(0,\xi ,y\right)=\varphi (\xi -\ln\left(\frac{x}{y}\right),\frac{\ln(\frac{x}{y})+\xi }{2}) \end{array}\right.$$

From Equation (33), we have

(38) $$\frac{\partial u(z;t,x,y)}{\partial x^{2}}=x^{-2}E\left[\frac{\partial \varphi }{\partial x^{2}}+\frac{\partial \varphi }{\partial x}\right]$$

Because of the function $\varphi \left(t,x\right)=\frac{1}{\sqrt{2\pi t}}e^{-\frac{x^{2}}{2t}}$, we have

(39) $$\frac{\partial \varphi }{\partial t}=\frac{1}{2}\frac{\partial \varphi }{\partial x^{2}}$$

Substituting Equations (38) and (39) into Equation (37), we obtain

(40) $$u_{t}\left(z;t,x,y\right)+\frac{1}{2}\sigma^{2}\left(y\right)P^{1-q}x^{2}u_{xx}\left(z;t,x,y\right)+\frac{v_{y}\left(t\right)}{1-V_{y}\left(t\right)}\int_{\Upsilon }P\left(y,dz\right)\left[u\left(t,x,z\right)-u\left(t,x,y\right)\right]=0$$

Hence, Equation (24) is the solution to Equation (23). ☐

**Theorem** **1.** The optimal risk-minimizing hedging portfolio strategy $\varphi^{*}\left(t\right)=\left(\alpha_{t}^{*},\beta_{t}^{*}\right)$ of the model in Equation (14) is

(41) $$\alpha_{t}^{*}=h_{x}\left(t,S_{t},Y_{t}\right)$$

(42) $$\beta_{t}^{*}=E\left(g\left(S_{t}\right)|F_{t}\right)-\alpha_{t}^{*}S_{t}$$

*where*

(43) $$g\left(S_{t}\right)=H={\left(S\left(t\right)-K\right)}^{+}$$

(44) $$E\left(g\left(S_{t}\right)|F_{t}\right)=E^{*}g\left(S_{t}\right)+\int_{0}^{t}h_{x}\left(v,S_{v},Y_{v}\right)dS_{v}+\int_{0}^{t}\int_{\Upsilon }\left(h\left(v,S_{v},y\right)-h\left(v,S_{v},Y_{v-}\right)\right)\left(\lambda -\tilde{\lambda }\right)\left(dv,dy\right)$$

(The Föllmer–Schweizer decomposition of the contingent claim $g\left(S_{t}\right)=H={\left(S\left(t\right)-K\right)}^{+}$.)

Under the optimal risk-minimizing hedging portfolio strategy $\varphi^{*}\left(t\right)$, the residual risk process in Equation (13) can be given by

(45) $$R_{t}\left(\varphi^{*}\right)=E\left[\int_{t}^{T}\left[\frac{v_{y}\left(t\right)}{1-V_{y}\left(t\right)}\int_{\Upsilon }P\left(y,dz\right)\left[h^{2}\left(s,S_{s},z\right)-h^{2}\left(t,x,y\right)\right]+2u_{t}\left(z;t,x,y\right)\right]\right]$$

**Proof** **of Theorem 1.** By Lemma 1, we know

(46) $$g\left(S_{T}\right)=h\left(T,S_{T},Y_{T}\right)$$

Using the It$\bar{o}$ formula, we have

(47) $$\begin{array}{c} g\left(S_{T}\right)=h\left(0,x,y\right)+\int_{0}^{T}h_{x}\left(v,S_{v},Y_{v}\right)dS_{v}+\int_{0}^{T}[h_{t}\left(v,S_{v},Y_{v}\right)+\\ \frac{1}{2}\sigma^{2}\left(Y_{v}\right)S_{v}^{2}P^{1-q}h_{xx}\left(v,S_{v},Y_{v}\right)]dv+\sum_{v\leq T}\left[h\left(v,S_{v},Y_{v}\right)-h\left(v-,S_{v-},Y_{v-}\right)\right] \end{array}$$

since the function $h(t,S_{t},Y_{t})$ is continuous over ([0,T]×Υ). It is right-continuous and left-limit too (see [19,20]). Hence, we can write the last part of Equation (47) as follows

(48) $$\begin{array}{c} \sum_{v\leq T}\left[h\left(v,S_{v},Y_{v}\right)-h\left(v-,S_{v-},Y_{v-}\right)\right]=\\ \int_{0}^{T}\int_{\Upsilon }\left(h\left(v,S_{v},y\right)-h\left(v-,S_{v-},Y_{v-}\right)\right)\lambda \left(dv,dy\right)\\ =\int_{0}^{T}\int_{\Upsilon }\left(h\left(v,S_{v},y\right)-h\left(v-,S_{v-},Y_{v-}\right)\right)\left(\lambda -\tilde{\lambda }\right)\left(dv,dy\right)+\\ \int_{0}^{T}\int_{\Upsilon }\left(h\left(v,S_{v},y\right)-h\left(v-,S_{v-},Y_{v-}\right)\right)\tilde{\lambda }\left(dv,dy\right)\\ =\int_{0}^{T}\int_{\Upsilon }\left(h\left(v,S_{v},y\right)-h\left(v-,S_{v-},Y_{v-}\right)\right)\left(\lambda -\tilde{\lambda }\right)\left(dv,dy\right)+\\ \int_{0}^{T}\int_{\Upsilon }\frac{v_{Y_{v-}}\left(v\right)}{1-V_{Y_{v-}}\left(v\right)}P\left(Y_{v-},dy\right)\left[h\left(v,S_{v},y\right)-u\left(v-,S_{v-},Y_{v-}\right)\right]dv \end{array}$$

Substituting Equation (48) into Equation (47), we can obtain

(49) $$\begin{array}{c} g\left(S_{T}\right)=h\left(0,x,y\right)+\int_{0}^{T}h_{x}\left(v,S_{v},Y_{v}\right)dS_{v}+\int_{0}^{T}[h_{t}\left(v,S_{v},Y_{v}\right)+\\ \frac{1}{2}\sigma^{2}\left(Y_{v}\right)S_{v}^{2}P^{1-q}h_{xx}\left(v,S_{v},Y_{v}\right)]dv+\\ \int_{0}^{T}\int_{\Upsilon }\left(h\left(v,S_{v},y\right)-h\left(v-,S_{v-},Y_{v-}\right)\right)\left(\lambda -\tilde{\lambda }\right)\left(dv,dy\right)+\\ \int_{0}^{T}\int_{\Upsilon }\frac{v_{Y_{v-}}\left(v\right)}{1-V_{Y_{v-}}\left(v\right)}P\left(Y_{v-},dy\right)\left[h\left(v,S_{v},y\right)-u\left(v-,S_{v-},Y_{v-}\right)\right]dv \end{array}$$

Combining the third and fifth formulas of Equation (49), we have

(50) $$\begin{array}{c} g\left(S_{T}\right)=h\left(0,x,y\right)+\int_{0}^{T}h_{x}\left(v,S_{v},Y_{v}\right)dS_{v}+\\ \int_{0}^{T}\int_{\Upsilon }\left(h\left(v,S_{v},y\right)-h\left(v-,S_{v-},Y_{v-}\right)\right)\left(\lambda -\tilde{\lambda }\right)\left(dv,dy\right)+\\ \int_{0}^{T}[h_{t}\left(v,S_{v},Y_{v}\right)+\frac{1}{2}\sigma^{2}\left(Y_{v}\right)S_{v}^{2}P^{1-q}h_{xx}\left(v,S_{v},Y_{v}\right)+\\ \int_{\Upsilon }\frac{v_{Y_{v-}}\left(v\right)}{1-V_{Y_{v-}}\left(v\right)}P\left(Y_{v-},dy\right)\left[h\left(v,S_{v},y\right)-u\left(v-,S_{v-},Y_{v-}\right)\right]]dv \end{array}$$

Using Equation (23), we know that in Equation (50) the value of the last formula is zero. That is, Equation (50) becomes

(51) $$g\left(S_{T}\right)=h\left(0,x,y\right)+\int_{0}^{T}h_{x}\left(v,S_{v},Y_{v}\right)dS_{v}+\int_{0}^{T}\int_{\Upsilon }\left(h\left(v,S_{v},y\right)-h\left(v-,S_{v-},Y_{v-}\right)\right)\left(\lambda -\tilde{\lambda }\right)\left(dv,dy\right)$$

Furthermore, Equation (51) is the Föllmer–Schweizer decomposition of the European call option payment $g\left(S_{t}\right)=H={\left(S\left(t\right)-K\right)}^{+}$. Hence, using Equations (21) and (22), we can obtain Equations (41) and (42). ☐

## 5. Summary

We propose an asset price model and consider a hedging problem for European call option. In the model, to accurately depict the price changes of risky assets, we employ a stochastic process derived from the non-extensive statistical theory to replace the classical geometric Brownian motion. Moreover, we embed a semi-Markov process into the model so that it can be developed to portray the macroeconomic impact. The regime-switching asset price model is an interesting topic. As future work, we will do further empirical research and option pricing problems under the model.
